# Quantitative Pharmacodynamic Characterization of Resistance versus Heteroresistance of Colistin in E. coli Using a Semimechanistic Modeling of Killing Curves

**DOI:** 10.1128/aac.00793-22

**Published:** 2022-08-30

**Authors:** Andrew Mead, Pierre-Louis Toutain, Pascal Richez, Ludovic Pelligand

**Affiliations:** a Comparative Biomedical Sciences, The Royal Veterinary College, London, United Kingdom; b TransPharm, Saint-Genies des Mourgues, France; c INTHERES, Université de Toulouse, INRAE, ENVT, Toulouse, France; d Clinical Services and Sciences, The Royal Veterinary College, London, United Kingdom

**Keywords:** colistin, heteroresistance, pharmacokinetics, pharmacodynamics, PK/PD modeling, polymyxin

## Abstract

Heteroresistance corresponds to the presence, in a bacterial isolate, of an initial small subpopulation of bacteria characterized by a significant reduction in their sensitivity to a given antibiotic. Mechanisms of heteroresistance versus resistance are poorly understood. The aim of this study was to explore heteroresistance in *mcr*-positive and *mcr*-negative Escherichia coli strains exposed to colistin by use of modeling killing curves with a semimechanistic model. We quantify, for a range of phenotypically (susceptibility based on MIC) and genotypically (carriage of *mcr*-1 or *mcr*-3 or *mcr*-negative) different bacteria, a maximum killing rate (*E*_max_) of colistin and the corresponding potency (EC_50_), i.e., the colistin concentrations corresponding to *E*_max_/2. Heteroresistant subpopulations were identified in both *mcr*-negative and *mcr*-positive E. coli as around 0.06% of the starting population. Minority heteroresistant bacteria, both for *mcr*-negative and *mcr*-positive strains, differed from the corresponding dominant populations only by the maximum killing rate of colistin (differences for *E*_max_ by a factor of 12.66 and 3.76 for *mcr*-negative and *mcr*-positive strains, respectively) and without alteration of their EC_50_s. On the other hand, the resistant *mcr*-positive strains are distinguished from the *mcr*-negative strains by differences in their EC_50_, which can reach a factor of 44 for their dominant population and 22 for their heteroresistant subpopulations. It is suggested that the underlying physiological mechanisms differ between resistance and heteroresistance, with resistance being linked to a decrease in the affinity of colistin for its site of action, whereas heteroresistance would, rather, be linked to an alteration of the target, which will be more difficult to be further changed or destroyed.

## INTRODUCTION

Heteroresistance is broadly defined by a heterogenous bacterial population comprising one or several subpopulations exhibiting decreased susceptibility to an antimicrobial compared to the main population (1). This phenomenon has been described for multiple antimicrobial drugs (AMDs) and is especially common for polymyxins (polymyxin B and colistin), where it has been reported against various species, including Escherichia coli (2, 3). Heteroresistant subpopulations, when exposed to AMD, may be enriched and result in a clinical challenge and treatment failure if concentrations in the biophase fail to reach inhibitory levels. Identifying and quantifying heteroresistance are challenging, with various methods, such as population analysis profiling (PAP), being labor intensive and limited based on MIC measurement (4). Previous studies have explored the use of time-kill curve (TKC) analysis coupled with PAP to explore the nature of heteroresistance (5, 6), although more advanced modeling of TKC is required to truly characterize heteroresistance.

Colistin’s antimicrobial effect has been previously reported as being concentration dependent against Acinetobacter baumannii (7, 8), Klebsiella pneumoniae (9), and Pseudomonas aeruginosa (10, 11). These studies have also described a major initial killing rate against colistin-susceptible strains, with bacterial regrowth observed at clinically relevant concentrations in static time-kill studies. The effect of colistin on E. coli has been explored in several studies using fixed pharmacological concentrations achievable in plasma (12) or urine (13), or in line with the epidemiological cutoff (ECOFF) of 2 mg/L (14). While these studies demonstrate the bactericidal activity of colistin and the synergistic effects of various drug combinations to potentiate the activity against resistant isolates, they have not attempted to describe the underlying pharmacodynamic (PD) parameters that control this bactericidal activity. Furthermore, studies that have included both susceptible and *mcr*-1-positive strains did not explore the influence of resistant genes on these key PD parameters (15). As for all drugs, the PD activity of AMD, which is generally reported by the measurement of MICs, can, in fact, be described in terms of its primary PD parameters of efficacy, potency, and sensitivity (16). For an AMD tested *in vitro*, efficacy will typically correspond to the maximal bactericidal effect. It is quantified by a maximum killing rate (*E*_max_; units per h). The potency is quantified by a concentration termed EC_50_ (unit mg/L), which is the AMD concentration for which a killing rate equal to *E*_max_/2 is obtained. The sensitivity relates to the more or less shallow or steep slope of the concentration versus effect response. It translates the more or less large extent of the concentrations having an action on the bacterial population from a minimal action to a maximal effect. This slope is measured by adding to the basic *E*_max_ model an additional parameter denoted here gamma (a scalar) to give the Hill model (17). In this context of the identification and quantification of PD parameters, the MIC must be viewed as a simple hybrid variable resulting from these parameters and which is observable under some standardized test conditions and at a single time point fixed between 18 and 20 h. TKC experiments, taking into account the time course of the activity of the AMD on the dynamics of growth, mortality, and possible regrowth of the tested bacterial population, provide essential information on underlying PD parameters. They can even be quantified from these TKCs with models predicting the temporal dynamics of the AMD effect, with these models being explicitly parameterized in terms of *E*_max_, EC_50_, and gamma. Several classes of models have been proposed for this purpose, in particular, the so-called semimechanistic models (18), which we have taken up in this article.

This investigation explores the antimicrobial effect of colistin against 7 different strains of E. coli (3 *mcr*-negative with low MICs; 3 *mcr*-1 positive and 1 *mcr*-3 positive with MICs at or above the ECOFF) by using static time-kill analysis with starting inoculum size of 10^5^ CFU/mL in cation-adjusted Mueller-Hinton broth (CAMHB). The aim was to quantify and compare pharmacokinetic (PK)/PD parameters describing the time course of growth and kill in these populations. The ultimate goal was to identify whether the overall model would provide insight into (i) differences between colistin efficacy and potency to different strains of E. coli, (ii) differences between *mcr*-negative and *mcr*-positive isolates (for example, growth-related fitness costs), (iii) the extent to which heteroresistance can explain the regrowth of bacteria with constant colistin concentration, and (iv) if the mechanisms underlying heteroresistance and resistance are qualitatively different or, on the contrary, of the same nature, if their expression is quantitatively different. We also present the first colistin time-kill assay, to our knowledge, performed with *mcr*-3-positive E. coli strains.

## RESULTS

### Time-kill assays.

Colistin time-kill curves for seven E. coli strains at an initial inoculum of 5 × 10^5^ CFU/mL were performed at multiples of the measured MICs (0×, 0.25×, 0.5×, 0.75×, 1×, 1.5×, 2×, and 4×) (Mead et al. [19]) and are shown as the geometric mean of three replicates as follows: 12241, N100, 219, 13846, 73h_B6_2, 120h_B3_5, and 2013-SQ352 (https://rvc-repository.worktribe.com/output/1567805/supplementary-data-quantitative-pharmacodynamic-characterization-of-resistance-vs-heteroresistance-of-colistin-in-e-coli-using-a-semi-mechanistic-modelling-of-killing-curves).

Visual inspection of the curves showed that all strains were capable of exponential growth in the absence of colistin, with a lag phase apparent in all replicates for isolates 12241, N100, and 219 and a single replicate for 13846. All strains reached a similar maximal bacterial population (stationary phase) at a geometric mean density of 3.01 × 10^10^ CFU/mL (range, 1.2 × 10^10^ to 5.4 × 10^10^). A rapid bactericidal (≥3 log reduction) effect against all isolates was observed, with bacterial kill as early as 10 min. The rate of kill was reduced in *mcr*-positive isolates, with the rate being concentration dependent compared to *mcr-*negative isolates. Regrowth was observed in all isolates at concentrations of ≤1× MIC.

### (i) Sequential TKC experiments.

**FIG 1:**
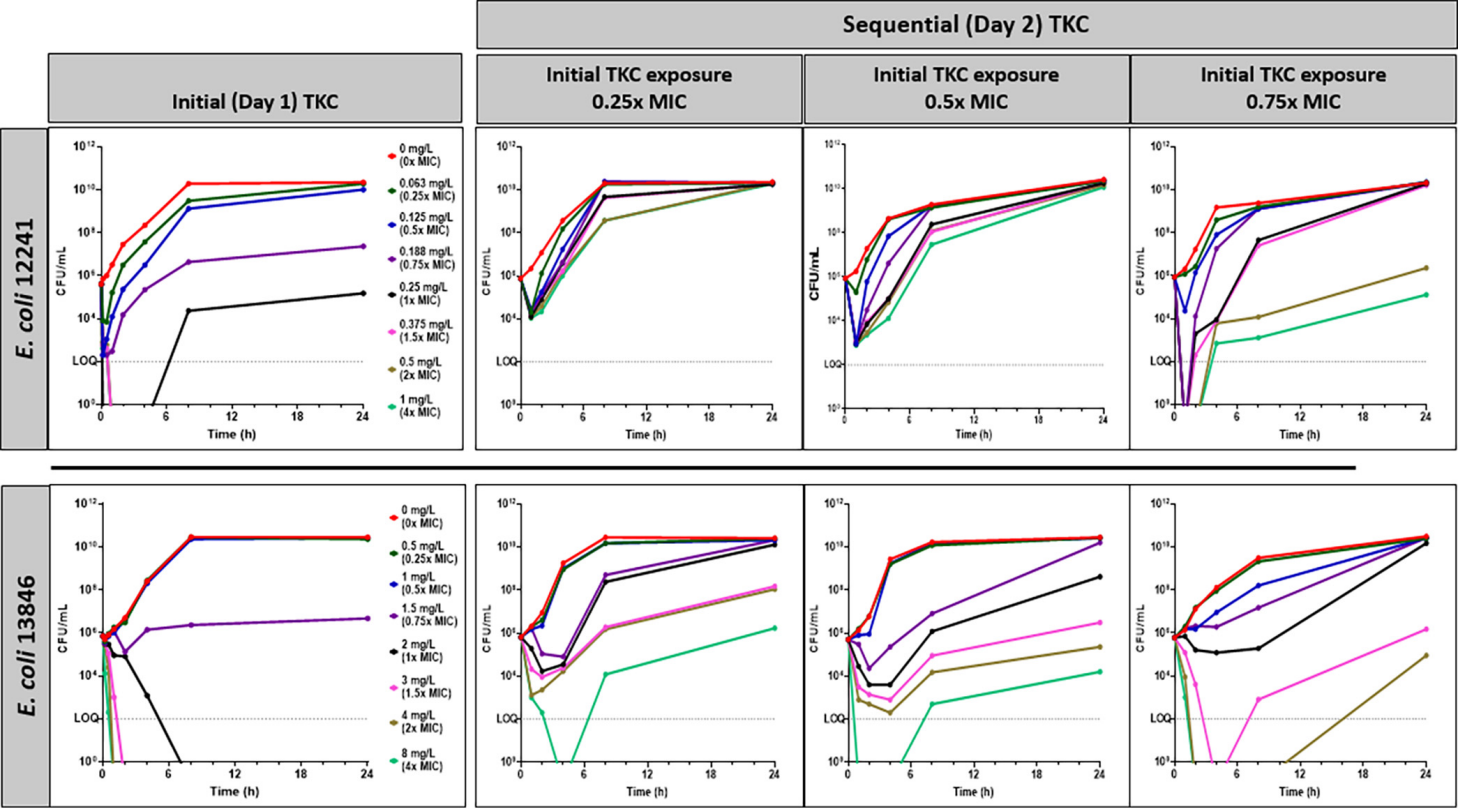
Sequential TKC experiments were performed and are presented as follows. (Top) NCTC reference E. coli strain 12241 (*mcr*-negative, MIC of 0.25 mg/L) showing initial TKC (day 1; column 1), and sequential (day 2) TKCs following exposure to 0.25× MIC (0.0625 mg/L; column 2), 0.5× MIC (0.125 mg/L; column 3), and 0.75× (0.188 mg/L; column 4) of the initial MIC. (Bottom) NCTC reference E. coli strain 13846 (*mcr*-1-positive, MIC of 2 mg/L) showing initial TKC (day 1; column 1), and sequential (day 2) TKC experiments following exposure to 0.25× MIC (0.5 mg/L; column 2), 0.5× MIC (1 mg/L; column 3), and 0.75× (1.5 mg/L; column 4) of the initial MIC. Repeated measurement of the MIC showed an increase of the MIC from 0.25 mg/L to 2 mg/L for isolate 12241 and 2 mg/L to 4 mg/L for isolate 13846 following the preexposure to colistin in the initial TKC, and this increase was irrespective of the preexposure concentration in the initial TKC.

Consecutive TKC experiments were performed with *mcr-*negative isolate 12241 and *mcr*-positive isolate 13846. The purpose of these sequential TKC experiments, with bacteria resulting from the regrowth of those previously exposed to different levels of colistin during day 1, was to explore a possible preconditioning effect on their sensitivity to colistin at the end of day 1 and during day 2. Rapid CFU decline followed by regrowth was observed for both isolates during the first TKC, at colistin concentrations up to 0.25 mg/L (1× MIC) for 12241 and 1.5 mg/L (0.75× MIC) for 13846 (Fig. 1). The second TKC experiment showed decreased susceptibility to colistin with regrowth observable up to 1 mg/L (4× MIC at the end of day 1) for 12241 and 4 mg/L (2× MIC at the end of day 1) for 13846, although with an initial kill rate in line with the concentration of colistin exposure in the first TKC experiment. The MICs increased for both isolates during the second TKC experiment, with isolate 12241 having a pre-TKC MIC (time zero h) of 0.25 mg/L and an MIC at 2 mg/L at time 24 h (after initial TKC experiment) and time 48 h (after secondary TKC); isolate 13846 had a pre-TKC MIC (time 0 h) of 2 mg/L and an MIC of 4 mg/L at time 24 h (after the initial TKC experiment) and at time 48 h (after the secondary TKC experiment). This indicates that the populations of bacteria selected during the regrowth at the end of day 1 have a reduced sensitivity to colistin that is evidenced by the second TKC experiment.

### PAP analysis.

**FIG 2:**
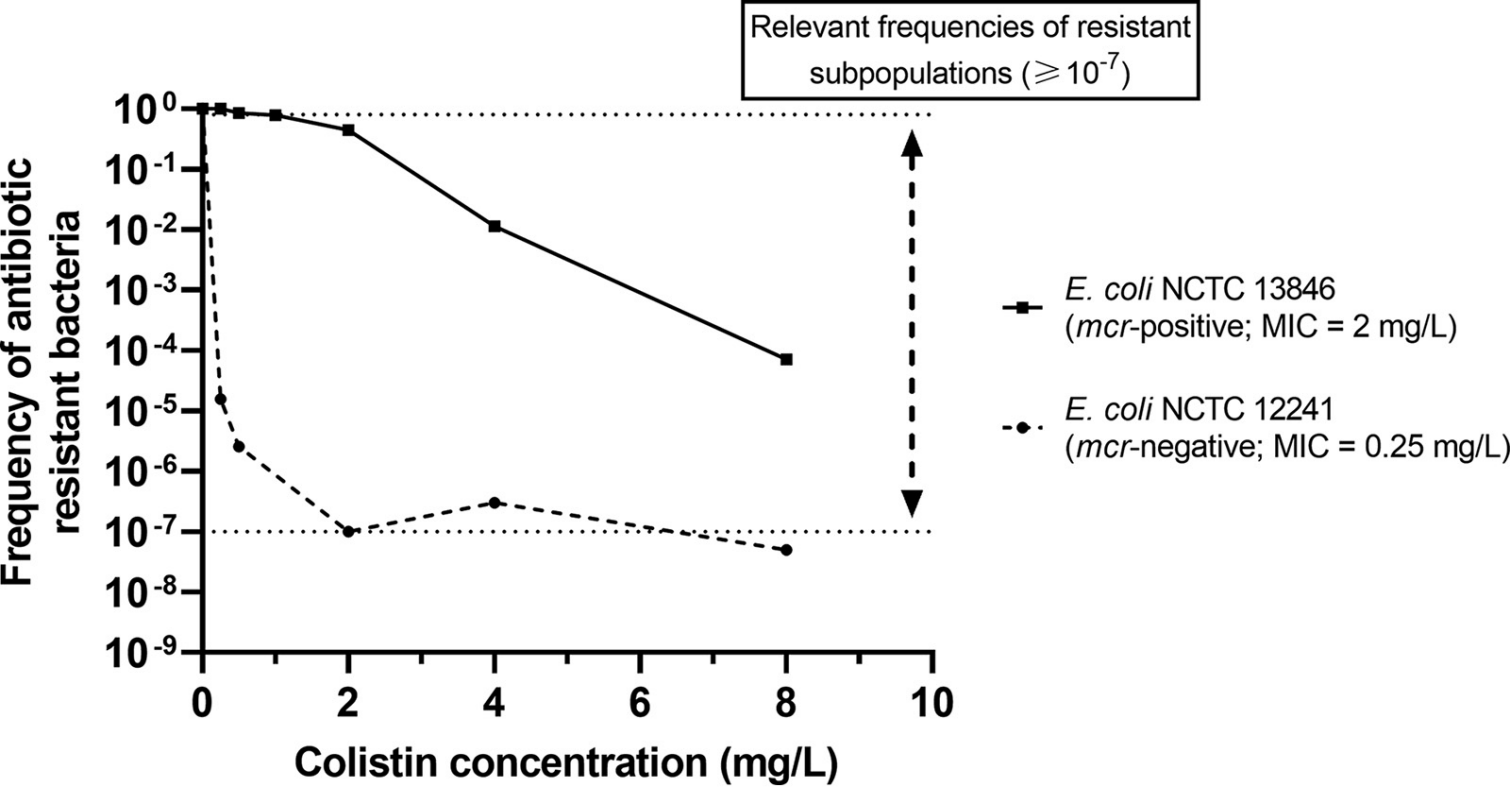
Population analysis profiling (PAP) of NCTC reference E. coli strains 13846 (*mcr*-1-positive, MIC of 2 mg/L) and 12241 (*mcr*-negative, MIC of 0.25 mg/L). Growth of colonies at greater than the MIC, including at concentrations ≥4-fold, indicates the presence of less susceptible subpopulations that suggest heteroresistance within the total bacterial population. Relevant frequencies that indicate heteroresistance are highlighted as defined by Andersson et al. (1).

The two tested isolates, *mcr*-negative isolate 12241 and *mcr*-positive isolate 13846, showed heteroresistant subpopulations recoverable up to 8 mg/L colistin base. The frequency of heteroresistant subpopulations, as a proportion of the total population determined in the absence of colistin, ranged from 1.02 × 10^−7^ to 3.32 × 10^−7^ in E. coli isolate 12241 (from 4 mg/L and 8 mg/L respectively) and 7.13 × 10^−5^ for E. coli isolate 13846 at 8 mg/L (Fig. 2).

### PD modeling of time-kill experiments.

**FIG 3:**
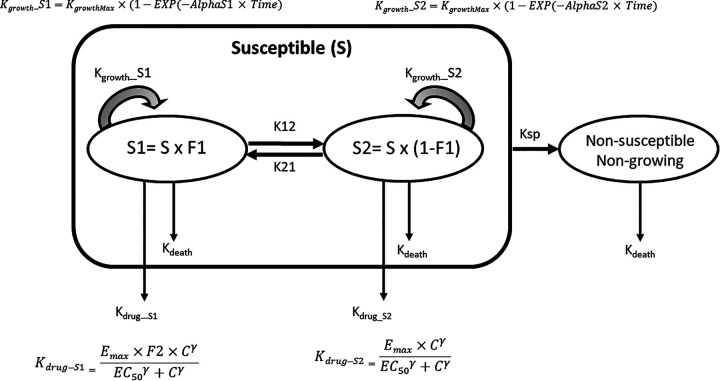
PK/PD model for colistin time-kill analysis. All bacteria were assumed to be in a susceptible (S) growing population from initial inoculation, with transfer to a nongrowing and nonsusceptible population. The starting susceptible bacterial population was considered to be heterogenous with two subpopulations, representing a dominant population of highly susceptible bacteria (S1) and a small population of less susceptible bacteria (S2) with a dynamic equilibrium between the two subpopulations driven by first-order rate constants of K12 and K21. The same maximal growth rate (*K*_growth_) was considered for S1 and S2, but the rate to achieve this maximal growth rate (alpha) was different for S1 and S2 (AlphaS1 for S1 and AlphaS2 for S2). The system was considered to be already in equilibrium during the initial exposure to colistin, and it is the K12/K21 ratio that was estimated by directly evaluating a distribution factor (F1) of the population between the population S1 (F1) and subpopulation S2 (1 − F1) with F1 = 1 − (K12/K21). The irreversible transfer rates to a nongrowing state (*K*_SP_) were considered identical for S1 and S2 All bacteria were subject to a constant rate of natural cell death (*K*_death_) fixed to 0.17 h^−1^. The effect of colistin (*K*_drug_), having the same dimension as *K*_death_ (h^−1^), was described by a Hill model with three parameters (*E*_max_ for efficacy, EC_50_ for potency, and gamma for the slope), with *C* being the colistin concentration at which bacteria are exposed. *K*_drug_ was considered additive to the natural cell death, with a potentiation factor (F2) for the highly susceptible subpopulation S1. F2 increases *K*_drug_ by only increasing *E*_max_ because in a preliminary analysis, it was shown there was no difference in EC_50_s for the two S1 and S2 subpopulations. A model script is given at https://rvc-repository.worktribe.com/output/1567805/supplementary-data-quantitative-pharmacodynamic-characterization-of-resistance-vs-heteroresistance-of-colistin-in-e-coli-using-a-semi-mechanistic-modelling-of-killing-curves.

The selected model is an adaptation of a generic semimechanistic model proposed by Nielsen et al. (20) (Fig. 3).

**FIG 4:**
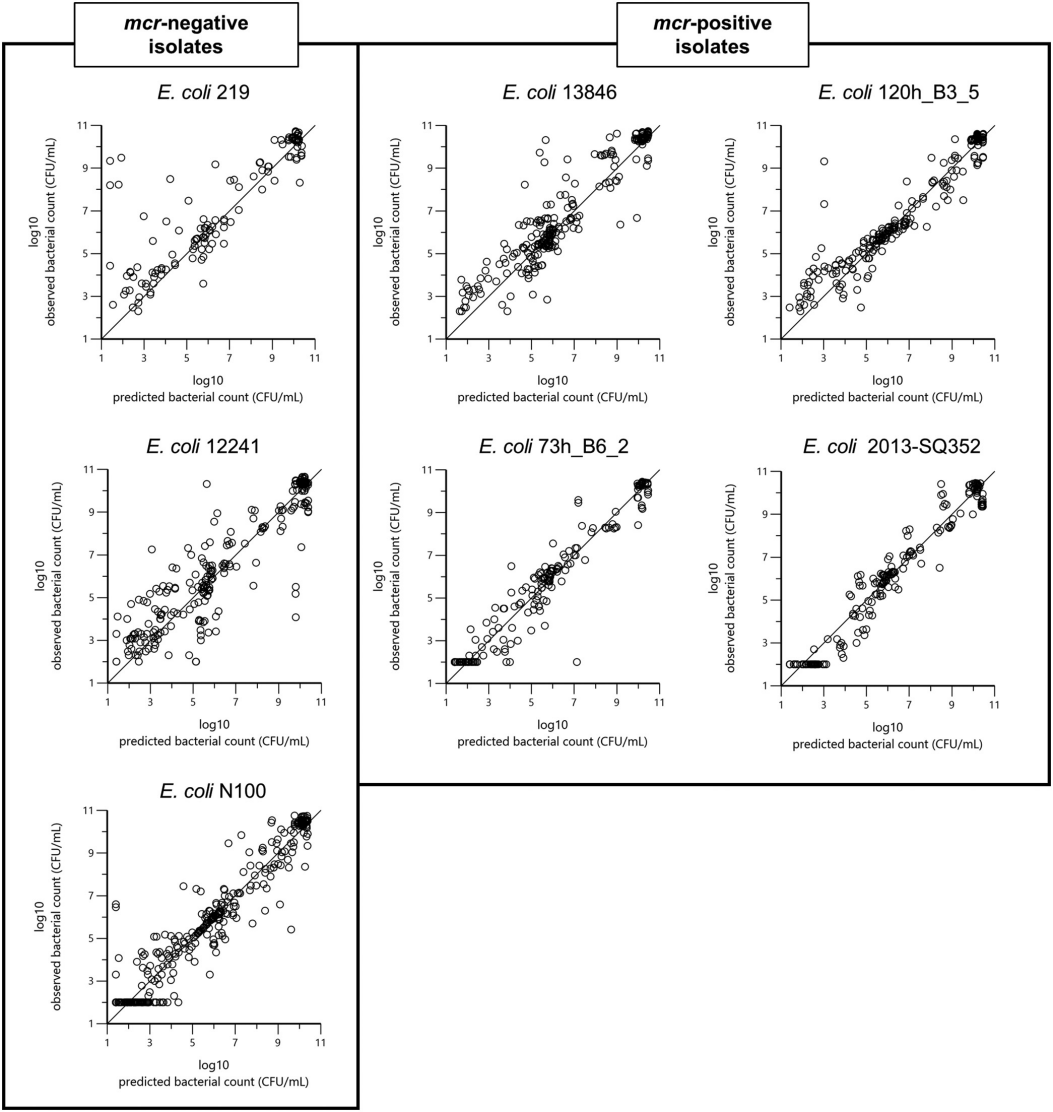
Plot of the observed natural logarithm of bacterial counts (CFU/mL; dependent variable [DV]) versus the log of individual predicted count values (PRED) for each of the seven strains. Ideally, observed versus fitted values should fall close to the line of unity, where *x* = *y*.

**FIG 5:**
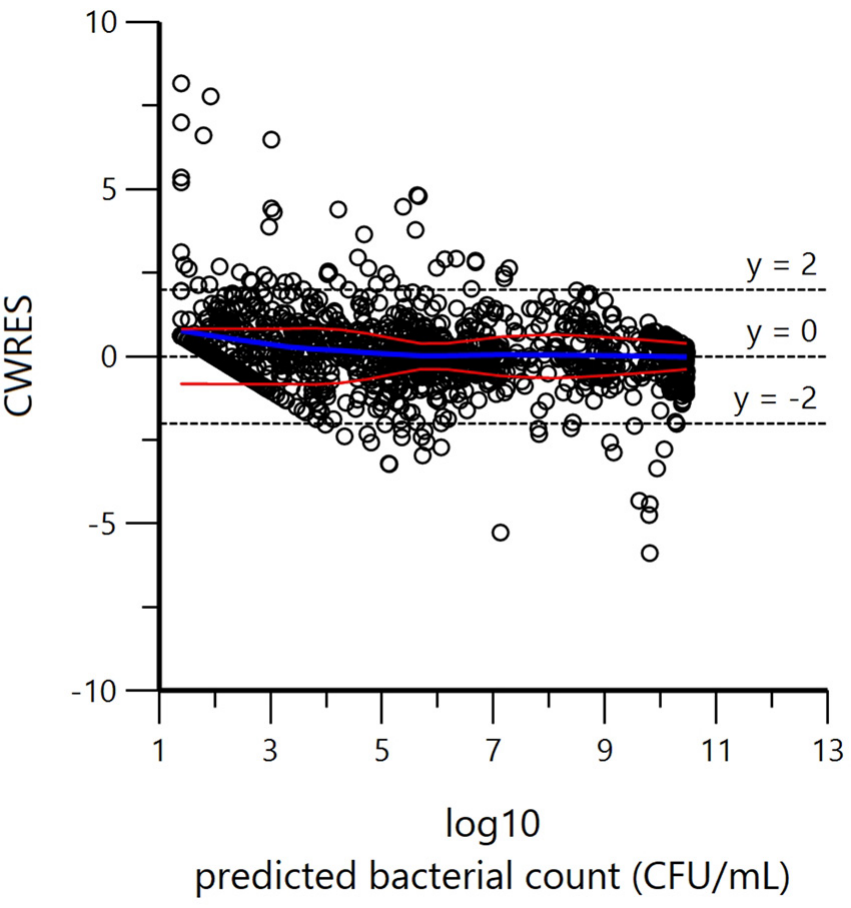
Conditional weighted residual (CWRES) versus PRED, where PRED is the individual prediction by the model (no random component in the model) indicating that the exponential residual error model was reasonable. Red and blue curves are LOcally wEighted Scatterplot Smoothing (LOESS) regression curves. The blue curve takes into account the sign of the residuals (positive or negative), while the red curve and its reflection only consider absolute values of residuals. Ideally, the blue line should be at 0, and the red line (with its negative reflection) should not show any fanning.

Goodness-of-fit plots (Fig. 4 and 5) indicate that the final selected model captured properly the different TKC experiments.

**FIG 6:**
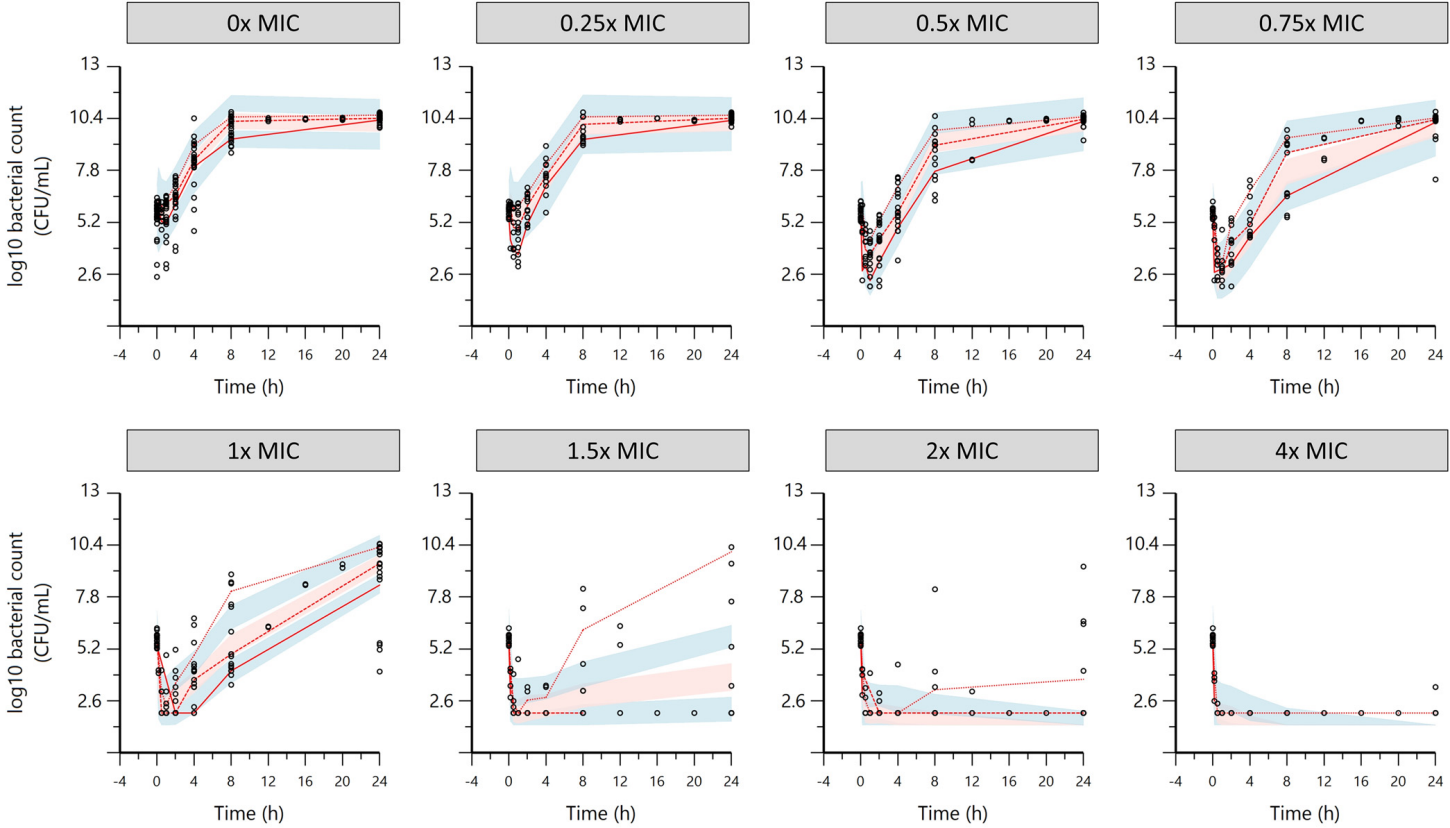
Visual predictive check (VPC) for the three *mcr*-negative E. coli isolates obtained with simulation of 200 replicates. The observed quantiles (20%, 50%, and 80%) (red lines) are reasonably well superimposed for most TKCs with the corresponding 90% confidence intervals (CIs; shaded area) of simulated data. Upper and lower blue shaded areas are the 90% CIs of 20 and 80% quantiles, respectively, and the pink shaded area is the 90% CI of the 50% quantile. Black open symbols, observed data.

**FIG 7:**
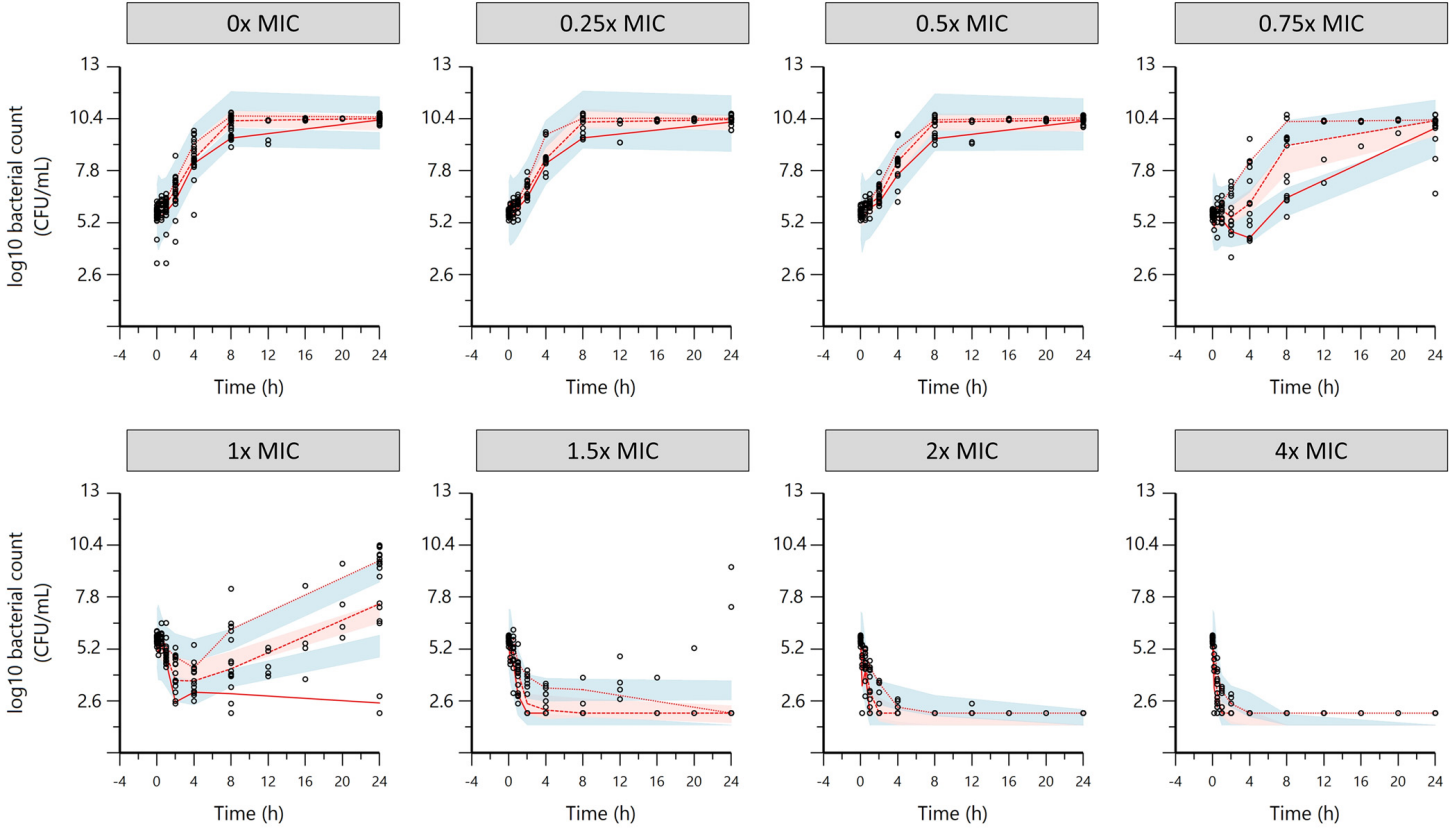
Visual predictive check (VPC) for the four *mcr*-positive E. coli isolates obtained with simulation of 200 replicates. The observed quantiles (20%, 50%, and 80%) (red lines) are reasonably well superimposed for most TKCs with the corresponding 90% confidence intervals (shaded area) of simulated data. Upper and lower blue shaded area are the 90% CI of 20 and 80% quantiles, respectively, and the pink shaded area is the 90% CI of the 50% quantile. Black open symbols, observed data.

Visual predictive checks (VPCs) for the *mcr*-negative strains and the *mcr*-positive strains are shown in Fig. 6 and 7, respectively.

Model-estimated typical values (30 samples, stratified by MCR status) are reported in text, with estimated bootstrap medians of parameters and their 95% confidence intervals presented in Table 1. The fractionation of the starting inoculum (F1) between S1 (the dominant and highly susceptible population) and S2 (the minority heteroresistant subpopulation) were slightly different between *mcr*-positive and *mcr*-negative isolates; with a starting inoculum of 5 × 10^5^ CFU/mL, estimates of numbers of heteroresistant bacteria were 269 and 363 for *mcr*-positive and *mcr*-negative isolates, respectively, i.e., about 0.06% of bacteria would be in subpopulations S2 at time zero. The maximal bacterial killing rate in the presence of colistin (*E*_max_) was not significantly different for the final S2 subpopulations of *mcr*-negative and *mcr*-positive strains and was estimated to be 2.69 h^−1^, i.e., a 15-fold increase compared to the death rate (fixed to 0.179 h^−1^).

**TABLE 1:** Typical value (tv) and median parameter estimates and 95% confidence intervals of the semimechanistic model describing TKC

Parameter^*a*^	Unit	Tv estimate	Median (bootstrap) estimate	%CI (from bootstrap)	
				2.5	97.5
Bacterial growth system parameters					
*K*_growthmax_	h^−1^	2.44	2.39	2.25	3.66
*K*_death_	h^−1^	0.179 (fixed)			
*B*_max_	CFU/mL	1.43 × 10^10^	1.49 × 10^10^	8.77 × 10^9^	1.98 × 10^10^
AlphaS1	h^−1^	0.88	0.99	0.38	1.29
AlphaS2	h^−1^	3.74	3.43	3.04	4.34
F1, *mcr* negative (no. of bacteria in S1 vs S2)		0.9995 (499,731 vs 269; 0.05%)	0.9996 (499,760 vs 240; 0.05%)	0.9992	0.9997
F1, *mcr* positive (no. of bacteria in S1 vs S2)		0.9994 (499,637 vs 363; 0.07%)	0.9994 (499,692 vs 308; 0.06%)	0.9989	0.9997
Colistin pharmacodynamic parameters					
EC_50__S1 (E. coli 219; *mcr* negative)	mg/L	0.082	0.084	0.046	0.098
EC_50__S1 (E. coli 12241; *mcr* negative)	mg/L	0.150	0.156	0.079	0.183
EC_50__S1 (E. coli N100; *mcr* negative)	mg/L	0.083	0.085	0.047	0.099
EC_50__S1 (E. coli 13486; *mcr*-1)	mg/L	2.935	2.744	2.664	3.499
EC_50__S1 (E. coli 120h_B3_5; *mcr*-1)	mg/L	2.760	2.624	2.340	3.408
EC_50__S1 (E. coli 73h_B6_2; *mcr*-1)	mg/L	3.669	3.683	3.369	4.442
EC_50__S1 (E. coli 2013_SQ352; *mcr*-3)	mg/L	3.370	3.150	2.908	4.069
EC_50__S2 (E. coli 219; *mcr* negative)	mg/L	0.114	0.114	0.105	0.157
EC_50__S2 (E. coli 12241; *mcr* negative)	mg/L	0.287	0.287	0.249	0.373
EC_50__S2 (E. coli N100; *mcr* negative)	mg/L	0.115	0.115	0.106	0.158
EC_50__S2 (E. coli 13486; *mcr*-1)	mg/L	1.462	1.500	1.255	1.751
EC_50__S2 (E. coli 120h_B3_5; *mcr*-1)	mg/L	2.174	2.063	1.907	3.634
EC_50__S2 (E. coli 73h_B6_2; *mcr*-1)	mg/L	2.540	2.530	2.213	3.005
EC_50__S2 (E. coli 2013_SQ352; *mcr*-3)	mg/L	2.063	2.017	1.907	2.977
Gamma_S1	Scalar	4.09	3.91	3.37	8.84
Gamma_S2	Scalar	3.22	3.80	1.79	8.38
F2 (for *mcr*-negative isolates)	Scalar	12.66	13.65	5.93	16.61
F2 (for *mcr*-positive isolates)	Scalar	3.76^*b*^	3.93^*b*^	1.72	6.13
*E*_max_ (S2) for both *mcr*-negative and *mcr*-positive isolates	h^−1^	2.69	2.51	2.04	5.29
*E*_max_ (S1) for *mcr*-negative isolates	h^−1^	34.12^*c*^	34.44^*c*^	28.61	38.44
*E*_max_ (S1) for *mcr*-positive isolates	h^−1^	10.13^*c*^	10.43^*c*^	7.58	13.66

a *K*_growthmax_, maximal growth rate constant; *K*_death_, natural death rate; *B*_max_, maximum possible bacterial density; alpha, delay before reaching maximal growth rate; F1, proportion of starting inoculum in subpopulation S1; EC_50,_ concentration required to achieve 50% of *E*_max_ as calculated according to equation 7 in (https://rvc-repository.worktribe.com/output/1567805/supplementary-data-quantitative-pharmacodynamic-characterization-of-resistance-vs-heteroresistance-of-colistin-in-e-coli-using-a-semi-mechanistic-modelling-of-killing-curves); γ_S1, Hill coefficient for subpopulation S1; γ_S2, Hill coefficient for subpopulation S2; F_2_, potentiation factor for *K*_drug_ in subpopulation S1; F2_MCR1_, potentiation factor for *K*_drug_ in subpopulation S1 for mcr-positive strains; *E*_max_, maximal increase in kill effect in addition to *K*_death_; tv, typical value.

b F2_MCR_ calculated from covariate parameter (dF2_MCR_) as F2_MCR_ = F2 × exp(dF2_MCR1_).

c *E*_max_ (S1) = *E*_max_ (S2) × F2.

The potentiation (F2) of the colistin effect on the highly susceptible subpopulation (S1) compared to the corresponding S2 was estimated at 12.66 for *mcr*-negative isolates, with a maximal killing rate of 34.12 per h for S1. In this condition, the time to eradicate S1, i.e., to achieve the limit of quantification (LOQ) of 100 CFU, was 15 min. For *mcr*-positive strains, the potentiation factor F2 was significantly lower at 3.76, giving a maximal killing rate for S1 of 10.13 per h. The eradication time of S1 for *mcr*-positive strains was 51 min. Colistin concentrations achieving half of the maximal effect (EC_50_) of the final S2 population were 0.114 to 0.287 mg/L for *mcr*-negative bacteria and 1.46 to 2.54 mg/L for *mcr*-positive bacteria, indicating a 10-fold-higher potency of colistin for *mcr*-negative bacteria. Furthermore, the resistant *mcr*-positive strains are distinguished from the *mcr*-negative strains by differences in their potency (EC_50_), which can reach a factor of 44 for their dominant population and 22 for their heteroresistant subpopulations. The maximal growth rates were not significantly different for the *mcr*-positive and *mcr*-negative strains (2.44 per h). However, the half-life to maximal growth rate constant (*K*_growthmax_) (time to reach 50% of *K*_growthmax_) was significantly longer for the S2 subpopulation (47 min) than for the S1 subpopulation (17 min).

**FIG 8:**
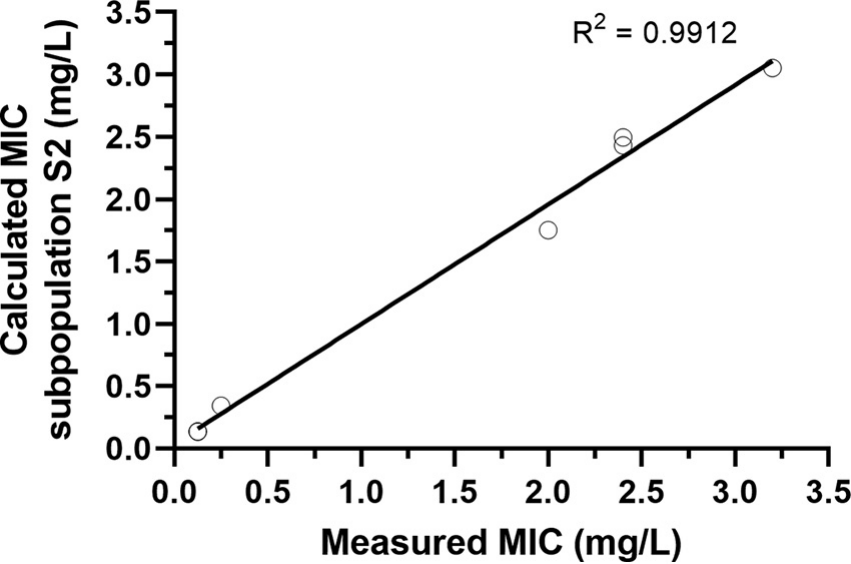
Estimated MIC for subpopulation S2 (as calculated from the PD model) shows good agreement with the MIC as measured by broth microdilution. The coefficient of determination (*R*^2^) is 0.9912.

MICs of the different subpopulations for each of the strains were computed as secondary parameters (Table 2). Model-calculated S2 MICs were very close to MICs measured by broth microdilution, suggesting that what is measured by the 24 h MIC is the MIC of the heteroresistant subpopulation. The model-estimated MICs of S1 and S2 subpopulations (Fig. 8) show that the estimated S2 MIC and the corresponding measured MIC show good agreement.

**TABLE 2:** MICs as estimated by the model

Secondary parameter	Unit	Data for:						
		*mcr*-negative E. coli isolates			*mcr*-1-positive E. coli isolates			*mcr*-3-positive E. coli isolates
		219	12241	N100	13846	120h_B3_5	73h_B6_2	2013_SQ352
MIC (as measured)	mg/L	0.125	0.25	0.125	2	2.4	3.2	2.4
MIC for subpopulation S1 (as calculated from the model^*a*^)	mg/L	0.041	0.076	0.042	2.096	1.940	2.566	2.304
MIC for subpopulation S2 (as calculated from the model^*b*^)	mg/L	0.136	0.342	0.137	1.751	2.495	3.051	2.430
Ratio of MICs of subpopulation S2 (calculated/measured)		1.09	1.37	1.10	0.88	1.04	0.95	1.01
Calculated fold difference of S2/S1		3.32	4.5	2.98	0.86	1.29	1.19	1.05

a MIC calculated based on (https://rvc-repository.worktribe.com/output/1567805/supplementary-data-quantitative-pharmacodynamic-characterization-of-resistance-vs-heteroresistance-of-colistin-in-e-coli-using-a-semi-mechanistic-modelling-of-killing-curves) (equation 13 or 14) with a standard inoculum of 5 × 10^5^ CFU/mL (46).

b MIC, MIC calculated based on (https://rvc-repository.worktribe.com/output/1567805/supplementary-data-quantitative-pharmacodynamic-characterization-of-resistance-vs-heteroresistance-of-colistin-in-e-coli-using-a-semi-mechanistic-modelling-of-killing-curves) (equation 12) with a standard inoculum of 5 × 10^5^ CFU/mL (46).

## DISCUSSION

In this study, the objective was to describe the antibacterial effect of colistin on strains of E. coli with different phenotypic and genetic susceptibility profiles, specifically differences between those that are *mcr* negative and have MICs within the susceptible wild-type population (ECOFF of 2 mg/L) and *mcr*-positive (*mcr*-1 or *mcr*-3) isolates with MICs beyond the ECOFF.

A rapid bactericidal effect as early as 10 min following the introduction of colistin, followed by bacterial regrowth in all but the highest colistin concentrations and across all strains regardless of *mcr* status, was observed. Bacterial regrowth has been recognized for a number of antibacterial drug classes, including in assays exploring the colistin bactericidal effect (21). Several reasons have been considered with regard to this phenomenon, including the loss of free colistin through binding to the assay system, transient adaptive resistance arising from exposure to colistin, and heterogeneity of susceptibility in the starting population (heteroresistance). The heteroresistance concept postulates that in any given bacterial population, even when described as sensitive to a given antibiotic, there will be a subpopulation that is less sensitive due to natural mutation, adaptation, or expression (1, 4). To determine the presence of heteroresistance within a population, two methods, population analysis profiling (PAP) and sequential TKC experiments, can be used. PAP is considered the gold-standard method, allowing the quantification of the frequency of less susceptible subpopulations through exposure to increasing antimicrobial concentrations (22, 23). PAP analysis of E. coli in the presence of colistin showed surviving bacterial colonies, for both *mcr-*negative and *mcr*-positive isolates, above the measured MICs and at 4-fold higher than ECOFFs, clearly indicating the presence of heteroresistance. This may be further supported by the sequential TKC experiment where a reduced susceptibility was observed after 24 h colistin exposure, at sub-MICs, and at subsequent increased MICs for both isolates. This may be indicative of a partial or complete reduction in the susceptible population and selection of less susceptible subpopulations from an initial heterogenous population.

Nicoloff et al. (3) suggested that up to 27.5% of bacteria-drug combinations may demonstrate heteroresistance, typically resulting from the duplication or amplification of a gene, leading to lower susceptibility, but which are unstable and will be lost/deleted, reverting to the normal wild type rapidly in the absence of any selective pressures. Multiple bacterial species, including E. coli, have been shown to demonstrate heteroresistance in the presence of colistin or polymyxin B (1, 24). Specifically, as colistin targets the lipopolysaccharide (LPS) on the outer membrane, variable phenotypic susceptibility is directly linked to heterogenicity in the phospholipid composition or as the result of altered regulation in the LPS modification pathways (2, 25, 26). Although not reported in this study, further clarification of heteroresistance may be possible through the tolerance disk test (TD test), a modification of the disk diffusion assay, proposed by Gefen et al. (27).

A semimechanistic model was developed for colistin as depicted in Fig. 3. A similar model structure was previously reported by Nielsen et al. (28) and adapted here for describing an initial heterogenous bacterial population in which a proportion (subpopulation, noted as S2) had a lower susceptibility than the dominant population, noted as S1. A reversible equilibrium between S1 and S2 was described by two first-order rate constants, noted as K12 and K21 in the model (see Fig. 3). With K12 and K21 being not separately identifiable, we assumed that the system was in an initial equilibrium between S1 and S2, with the total population being apportioned immediately prior to the onset of colistin exposure according to a factor noted as F1 that is actually the identifiable ratio K21/K12. This type of model makes it possible to describe, for each subpopulation, the temporal development of the response to the colistin exposure, including an initial phase of rapid killing followed by regrowth. In addition, it allows quantification of the basic pharmacodynamic parameters (efficacy, potency, and sensitivity of the relation concentration versus effect), which collectively will determine the MIC, which is measured at 24 h. In this perspective, it should be kept in mind that an MIC is only a hybrid variable whose value depends not only on the underlying PD parameters of the antibiotic but also on the conditions under which it is measured (initial inoculum load, test duration, bacterial growth rate). Our model estimated the initial less susceptible subpopulation being of about a few hundred bacteria, i.e. largely the minority, and that for both *mcr*-negative and *mcr*-positive populations, it demonstrated that heteroresistance may still be present in isolates harboring resistance genes. The reason why the least susceptible subpopulation, S2, remains very much in the minority in the presence of the most sensitive S1 subpopulation is not explained by the model. By considering our model with an equilibrium between the two subpopulations governed by K12 and K21, we can only hypothesize that the presence of the most sensitive subpopulation S1 exerts a double brake, on one hand, on K12, which remains much lower than K21, and on the other hand, on the *K*_growth_ rate of the least sensitive population, S2, that cannot replicate in the presence of the most sensitive one. However, the intrinsic growth rates of these two subpopulations (in the absence and in the presence of colistin, respectively) are of the same order of magnitude, but our model showed that the delay to achieve this maximal growth rate is shorter for S2 than S1 (as estimated by the parameter alpha), suggesting a lack of fitness cost regarding the growth rate of the S2 heteroresistant subpopulation.

For both *mcr*-negative and *mcr*-positive isolates, the initial bactericidal effect of colistin as estimated by the maximal killing rate was rather rapid (*mcr* positive) or very rapid (*mcr* negative) with average maximal killing times (1/*E*_max_) of 5.9 min and 1.8 min, respectively. This means that for a very large colistin concentration, the mean time required to kill a population of bacteria is only a few minutes for both *mcr*-negative and *mcr*-positive strains. It is this initial rapid killing that ensures the eradication of the most susceptible subpopulation, S1, that is rapidly replaced by the less susceptible subpopulation, S2, a phenomenon that is described as a regrowth but that is actually the growth of the initial S2 minority subpopulation. Finally, the MIC that is measured at 24 h is not the MIC of the initial S1 dominant subpopulation but the MIC of the S2 minority subpopulation.

The maximal killing rate is the pharmacological efficacy parameter, while EC_50_ is a measure of colistin potency. Efficacy and potency are two different descriptors of colistin effect. *E*_max_ reflects the property that enables colistin to produce a given response on the bacteria (here, the bacterial death). *E*_max_ is a proportionality factor that is related to some bacterial factor as the structure of cell wall or membrane integrity. Although modeled here using the Hill model (as previously reported by Bergen et al. [10]), it has been suggested that the self-uptake of colistin may also be described by a second-order kill rate (29); in our model, we can identify a linear effect (at least between 20 and 80% of the response in the log domain), and the Hill model correctly describes log-linear dose responses before predicting a saturation, which, considering the rapid kill effect, is unlikely to be reached. In contrast, EC_50_, which is always expressed as a concentration, reflect the colistin affinity for its target. The model indicates a major difference of EC_50_ between *mcr*-negative and *mcr*-positive strains but for a given *mcr-*negative or a *mcr*-positive strain, and subtle differences between subpopulations within a given strain, but it is observed that the maximal killing rate is the principle component that differentiated the two initial subpopulations. To estimate the effect of colistin on a heteroresistant subpopulation, a potentiation factor (F2) was included in the model to estimate the increase in *E*_max_, i.e., the maximum killing rate against the highly susceptible S1 subpopulation. For *mcr*-negative isolates, the colistin effect was 12.66 times more for the high-susceptibility S1 subpopulation. The model indicates that this potentiation factor was lower for *mcr*-harboring isolates, where the highest-susceptibility subpopulation, S1, was only 3.76-fold more susceptible than the minority S2 *mcr* subpopulation. This suggests that it is the nature of the colistin’s target that differentiates S1 from S2 and not the affinity of colistin for its site of action as a lower EC_50_ for S1 than the S2 subpopulation would suggest. This supports the hypothesis that the bactericidal effect of colistin in S1 is easier to achieve than the one of S2 for a given colistin concentration at the action site.

The potency (EC_50_) was individually fitted between strains, significantly improving the model fit compared to fitting a random distribution around a typical value for grouped strains, with individualized values being in line with the measured MICs for each isolate. Strain-specific potency has previously been illustrated by Nielsen et al. (28) to allow for model adaptation to strain variability. In addition, thanks to the estimation of EC_50_ and *E*_max_ specific to each subpopulation, it was possible to calculate, for each subpopulation, the corresponding MIC, in particular, that of S1, while only that of S2 is observable at 24 h. The MICs calculated for S2 were very similar to the MICs measured at 24 h (see Fig. 8), supporting the validity of the adopted model. In this respect, the measured and observed MICs depend not only on *E*_max_ and EC_50_ as previously discussed but also on the growth rates, which have been estimated separately for *mcr*-positive and *mcr*-negative bacteria. This allows discussion of a fitness cost that could be associated with the presence of an *mcr* gene. Indeed, if AMR mechanisms confer an advantage when in selective environments (here in the presence of colistin), this is often balanced by an inherent fitness cost (30, 31). The model did not indicate (through the inclusion of an *mcr*-related covariate on *K*_growthmax_ or alpha) a difference in maximal growth rate or lag phase attributable to an underlying fitness cost of MCR. This is consistent with the fact that estimations of the maximal bacterial population (*B*_max_) showed similar values across strains, although multiple studies have explored the potential fitness cost of MCR, reporting a substantial reduction in fitness attributed to the energetic cost of enzyme production and the cell membrane instability resulting from LPS modification (32–34). This fitness cost may not be universal across all strains, with some bacteria showing compensatory mutations (35). Without complete genetic profiling or further studies to elucidate the relative fitness of the strains in this study, the model did not indicate (through the inclusion of an *mcr*-related covariate on *K*_growthmax_) a difference in maximal growth rate attributable to an underlying fitness cost of MCR.

The main limit of our investigation is the possible confounding factor represented by a possible gradual, time-dependent loss of colistin through adsorption to assay materials, for example, the polystyrene assay plate, which may reduce the proportion of free colistin and may no longer exhibit an antibacterial effect (36, 37). The rate and extent of colistin adsorption are likely to be dependent on the specifics of the time-kill assay setup (e.g., brand of assay plate, media, etc.) and can only be accurately determined if colistin concentration is measured throughout the assay. In this study, there were no data on the possible binding rate of colistin. This may be partially mitigated by the measurement of MIC, which, as it is performed at a fixed time point of 24 h and in the same polystyrene microassay plate, already would take into consideration an amount of colistin binding. Furthermore, regrowth related to colistin adsorption alone would not account for the change in MIC recorded following colistin exposure (i.e., in the absence of heteroresistance MIC would be expected to remain the same).

### Conclusion.

In conclusion, this study explores the bactericidal effect of colistin against multiple strains of E. coli, and, through the development and implementation of a semimechanistic model, it determines the PK/PD parameters. Investigation of these parameters, when applied individually to strains harboring mobilized colistin resistance genes, indicates that differences in colistin potency and potentiation of the drug effect on the more susceptible proportion of a heteroresistant population are key to understanding how colistin treatment impacts these low-susceptibility strains. Our modeling approach suggests that heteroresistance (S1 versus S2) is related to the structure of the target, which, for a given concentration of colistin, will be more difficult to alter by colistin, while resistance is, rather, related to the need for higher colistin concentrations to act on a target which is of the same nature in sensitive (S2) and resistant bacteria (S2).

## MATERIALS AND METHODS

### Bacterial isolates.

Seven E. coli isolates were selected to cover a range of phenotypic resistance profiles based on MICs and their genetic profile if harboring mobilized colistin resistance. The selected isolates were (i) *mcr*-negative E. coli 12241 (NCTC reference strain), (ii) *mcr*-negative E. coli 219, (iii) *mcr*-negative E. coli N100, (iv) *mcr*-1-positive E. coli 13846 (NCTC reference strain), (v) *mcr*-1-positive E. coli 73h_B6_2, (vi) *mcr*-1-positive E. coli 120h_B3_5, and (vii) *mcr*-3-positive E. coli 2013-SQ352 as previously reported by Mead et al. (19).

Isolates were recovered from −80°C storage in MHB-glycerol stock, cultured on MacConkey agar (Oxoid, Basingstoke, UK), and stored on agar at 4°C for up to 7 days prior to further analysis.

### Colistin.

Two batches of European Pharmacopoeia-compliant Meiji Seika Pharma’s colistin sulfate (ColiMeiji, here “colistin”) were used in this study with a potency, as supplied (and reported by certificate of analysis), of (i) 23,558 IU/mg colistin sulfate supplied by Wyjolab (Chaillac, France), and (ii) 24,458 IU/mg colistin sulfate supplied by Dopharma (Saint-Herblon, France). Batches were considered equivalent when adjusted for colistin base equivalence. Colistin sulfate working solution was prepared immediately prior to dosing at a stock concentration of 2 × 10^6^ IU/mL colistin base.

### MICs.

MICs had previously been determined for each isolate using a 2-fold dilution series according to the broth microdilution method described in the European Committee for Antimicrobial Testing (EUCAST) guidelines and in accordance with ISO 20776 (38, 39). This method was adapted to 5-overlapping 2-fold dilution series to increase accuracy to within 20% of the dilution (compared to standard 2-fold dilution series) as previously described (40–42).

Bacterial suspensions were prepared from individual colonies suspended in phosphate-buffered saline (PBS) with comparison to 3 McFarland standards using DensiCheck Plus (bioMérieux, Hampshire, UK). Suspensions were diluted in CAMHB to achieve a final in-plate inoculum of 5 × 10^5^ CFU/mL. The MIC was recorded following overnight static incubation at 37°C. Two control isolates (*mcr*-1 negative [NCTC 12241] with an expected MIC of 0.5 or 1 mg/L and *mcr*-1 positive [NCTC 13846] with an expected MIC of 4 mg/L) were included in each plate, with MICs accepted within one dilution of the expected range.

### *In vitro* antimicrobial growth (time-kill) experiments.

Time-kill curve (TKC) assays were carried out at a minimum of triplicate over 3 days at a starting inoculum 5 × 10^5^ CFU/mL, with additional growth curves and TKC assays performed at various inoculum sizes to optimize subsequent analysis as outlined in (https://rvc-repository.worktribe.com/output/1567805/supplementary-data-quantitative-pharmacodynamic-characterization-of-resistance-vs-heteroresistance-of-colistin-in-e-coli-using-a-semi-mechanistic-modelling-of-killing-curves).

The bacterial isolates were cultured overnight on Mueller-Hinton (MH) agar. Up to 3 colonies were transferred into 5 mL of CAMHB and incubated (orbital shaker, 37°C) for 2 h prior to performing the TKC assay to promote optimal log-phase growth. The bacterial culture was diluted, using prewarmed CAMHB, to achieve a density of approximately 1 × 10^8^ CFU/mL compared to a 0.5 McFarland standard; this was diluted to achieve final in-plate counts of 5 × 10^5^ CFU/mL. Colistin was prepared at multiples of the MICs for each respective isolate, and TKC assays were run in 96-well plates, with each row representing a different concentration (0, 0.125, 0.25, 0.50, 0.75, 1, 1.5, 2, and 4× MIC) and each column a different time (0, 0.167, 0.5, 1, 2, 4, 8, 12, 16, 20, and 24 h postinoculation). Plates were incubated statically at 37°C.

As each well represents an individual sample point (time and MIC multiple), the entire contents (100 μL) were sampled and 10-fold serially diluted in PBS, covering the estimated range of the bacterial count. The spot-plate method utilized 10-μL spots on MacConkey agar, followed by static overnight incubation at 37°C, to allow for colony counts to be performed. Bacterial density (CFU/mL) was then back calculated using the dilution factor and spot volume. The limit of quantification (LOQ) was 100 CFU/mL (1 colony from 10-μL spot of undiluted sample).

### Sequential TKC experiments.

Consecutive TKC experiments were performed with *mcr-*negative isolate 12241 and *mcr*-positive isolate 13846. Initial TKC experiments were performed as previously described (43); at 24 h, bacteria that regrew to a large visible pellet (approximately1 × 10^8^ CFU/mL or higher) were harvested by centrifugation and resuspended at 1 × 10^5^ CFU/mL with 1 h growth before the start of the secondary TKC experiment. MICs were measured at 24 h (after the initial TKC experiment) and 48 h (after the secondary TKC experiment).

### Population analysis profile.

Analysis of colistin heteroresistant subpopulations of E. coli, *mcr-*negative isolate 12241 and *mcr*-positive isolate 13846, by population analysis profiles (PAPs) was conducted in duplicate by spread-plating 100 μL of the starting bacterial cell suspension (overnight bacterial culture) and its serial PBS dilutions on Mueller-Hinton agar plates (Oxoid, UK) with various concentrations (0, 0.25, 0.5, 1, 2, 4, and 8 mg/L) of base colistin. Colonies were counted after overnight incubation at 37°C. The frequency of less susceptible bacterial subpopulations was defined as the ratio of counts obtained on colistin-containing agar versus the total bacterial population in the absence of colistin after back calculation to density in undiluted (starting) culture.

### PD modeling of *in vitro* TKC experiments.

Pharmacodynamic data analyses were conducted using Phoenix NLME v8.3.0.5005 (Pharsight Corporation, St. Louis, MO, USA). Determination of the PD parameters from time-kill experiments of all 7 strains at starting target inoculums of 5 × 10^2^, 5 × 10^4^, 5 × 10^5^, and 5 × 10^6^ CFU/mL (see https://rvc-repository.worktribe.com/output/1567805/supplementary-data-quantitative-pharmacodynamic-characterization-of-resistance-vs-heteroresistance-of-colistin-in-e-coli-using-a-semi-mechanistic-modelling-of-killing-curves) were analyzed simultaneously using a semimechanistic PD model adapted from Nielsen and Friberg (18). The full model approach is described in (https://rvc-repository.worktribe.com/output/1567805/supplementary-data-quantitative-pharmacodynamic-characterization-of-resistance-vs-heteroresistance-of-colistin-in-e-coli-using-a-semi-mechanistic-modelling-of-killing-curves) and outlined below. A diagrammatic representation of the final model is given in Fig. 3.

To allow for parameter variation related to the categorical *mcr*, a covariate for strains harboring *mcr* (*mcr*-1 or *mcr-*3) versus non-*mcr* was included in the PD parameters (*E*_max_, gamma, *K*_growthmax_, alpha, *B*_max_, F1, and F2) of the model. A covariate search was run on the population model to evaluate all covariate combinations using the shotgun Phoenix tool.

Values below the limit of quantification (BLQ; ≤100 CFU/mL; 12.63% of the complete data set) were retained in the analysis by using a likelihood-based approach according to the M3 method (44).

Residual variability was modeled with an exponential error model (45). When a log-additive error model is specified, and if there is only one error model, the predictions and observations are log transformed by Phoenix and are fit into that space. Parameter estimates were based on minimizing an objective function value, using the Phoenix Naive pooled engine, that treats all observations as if they came from a single individual in that it ignores interindividual variations (no random components are computed), but the engine respects interindividual differences in initial conditions and covariate values. As this engine was not able to return percent coefficient of variation (%CV) of estimates (precision), the estimated median of fixed effect parameters (EC_50_, *E*_max_, alpha, gamma, *K*_growthmax_, *B*_max_, F1, F2, and all theta values of covariates) are reported in Table 1 as typical values with confidence intervals determined using a bootstrap method, while typical values of parameters obtained with the naive pool engine are reported in the text.

Adequacy of model fit was determined through the different diagnostic goodness-of-fit plots, including visual predictive check (VPC). VPCs were obtained by simulating 200 replicates to plot 20, 50, and 80% predictive check quantiles with their 90% confidence intervals.

Additional secondary parameters were calculated directly from the model, including MIC and minimal bactericidal concentrations (MBCs) as described by Mouton et al. (46).

## References

[1] Andersson DI, Nicoloff H, Hjort K. 2019. Mechanisms and clinical relevance of bacterial heteroresistance. Nat Rev Microbiol 17:479–496. 10.1038/s41579-019-0218-1. 31235888

[2] Juhász E, Iván M, Pintér E, Pongrácz J, Kristóf K. 2017. Colistin resistance among blood culture isolates at a tertiary care centre in Hungary. J Glob Antimicrob Resist 11:167–170. 10.1016/j.jgar.2017.08.002. 28838854

[3] Nicoloff H, Hjort K, Levin BR, Andersson DI. 2019. The high prevalence of antibiotic heteroresistance in pathogenic bacteria is mainly caused by gene amplification. Nat Microbiol 4:504–514. 10.1038/s41564-018-0342-0. 30742072

[4] El-Halfawy OM, Valvano MA. 2015. Antimicrobial heteroresistance: an emerging field in need of clarity. Clin Microbiol Rev 28:191–207. 10.1128/CMR.00058-14. 25567227 PMC4284305

[5] Li J, Rayner CR, Nation RL, Owen RJ, Spelman D, Tan KE, Liolios L. 2006. Heteroresistance to colistin in multidrug-resistant Acinetobacter baumannii. Antimicrob Agents Chemother 50:2946–2950. 10.1128/AAC.00103-06. 16940086 PMC1563544

[6] Pournaras S, Kristo I, Vrioni G, Ikonomidis A, Poulou A, Petropoulou D, Tsakris A. 2010. Characteristics of meropenem heteroresistance in Klebsiella pneumoniae carbapenemase (KPC)-producing clinical isolates of K. pneumoniae. J Clin Microbiol 48:2601–2604. 10.1128/JCM.02134-09. 20504985 PMC2897536

[7] Owen RJ, Li J, Nation RL, Spelman D. 2007. In vitro pharmacodynamics of colistin against Acinetobacter baumannii clinical isolates. J Antimicrob Chemother 59:473–477. 10.1093/jac/dkl512. 17289768

[8] Dudhani RV, Turnidge JD, Nation RL, Li J. 2010. f AUC/MIC is the most predictive pharmacokinetic/pharmacodynamic index of colistin against Acinetobacter baumannii in murine thigh and lung infection models. J Antimicrob Chemother 65:1984–1990. 10.1093/jac/dkq226. 20573659 PMC2920176

[9] Poudyal A, Howden BP, Bell JM, Gao W, Owen RJ, Turnidge JD, Nation RL, Li J. 2008. In vitro pharmacodynamics of colistin against multidrug-resistant Klebsiella pneumoniae. J Antimicrob Chemother 62:1311–1318. 10.1093/jac/dkn425. 18922815

[10] Bergen PJ, Bulitta JB, Forrest A, Tsuji BT, Li J, Nation RL. 2010. Pharmacokinetic/pharmacodynamic investigation of colistin against Pseudomonas aeruginosa using an in vitro model. Antimicrob Agents Chemother 54:3783–3789. 10.1128/AAC.00903-09. 20585118 PMC2934992

[11] Dudhani RV, Turnidge JD, Coulthard K, Milne RW, Rayner CR, Li J, Nation RL. 2010. Elucidation of the pharmacokinetic/pharmacodynamic determinant of colistin activity against Pseudomonas aeruginosa in murine thigh and lung infection models. Antimicrob Agents Chemother 54:1117–1124. 10.1128/AAC.01114-09. 20028824 PMC2826009

[12] Loose M, Naber KG, Hu Y, Coates A, Wagenlehner FM. 2018. Serum bactericidal activity of colistin and azidothymidine combinations against mcr-1-positive colistin-resistant Escherichia coli. Int J Antimicrob Agents 52:783–789. 10.1016/j.ijantimicag.2018.08.010. 30138665

[13] Loose M, Naber KG, Hu Y, Coates A, Wagenlehner FM. 2019. Urinary bactericidal activity of colistin and azidothymidine combinations against mcr-1-positive colistin-resistant Escherichia coli. Int J Antimicrob Agents 54:55–61. 10.1016/j.ijantimicag.2019.04.011. 31034939

[14] Cannatelli A, Principato S, Colavecchio OL, Pallecchi L, Rossolini GM. 2018. Synergistic activity of colistin in combination with resveratrol against colistin-resistant Gram-negative pathogens. Front Microbiol 9:1808. 10.3389/fmicb.2018.01808. 30131787 PMC6091244

[15] Zhou Y-F, Tao M-T, Feng Y, Yang R-S, Liao X-P, Liu Y-H, Sun J. 2017. Increased activity of colistin in combination with amikacin against Escherichia coli co-producing NDM-5 and MCR-1. J Antimicrob Chemother 72:1723–1730. 10.1093/jac/dkx038. 28333193

[16] Mouton JW, Muller AE, Canton R, Giske CG, Kahlmeter G, Turnidge J. 2017. MIC-based dose adjustment: facts and fables. J Antimicrob Chemother 73:564–568. 10.1093/jac/dkx427. 29216348

[17] Toutain P-L. 2002. Pharmacokinetic/pharmacodynamic integration in drug development and dosage-regimen optimization for veterinary medicine. AAPS PharmSci 4:160–188. 10.1208/ps040438. PMC2751327 12646010

[18] Nielsen EI, Friberg LE. 2013. Pharmacokinetic-pharmacodynamic modeling of antibacterial drugs. Pharmacol Rev 65:1053–1090. 10.1124/pr.111.005769. 23803529

[19] Mead A, Billon-Lotz C, Olsen R, Swift B, Richez P, Stabler R, Pelligand L. 2022. Epidemiological prevalence of phenotypical resistances and mobilised colistin resistance in avian commensal and pathogenic E. coli from Denmark, France, The Netherlands, and the UK. Antibiotics 11:631. 10.3390/antibiotics11050631. 35625275 PMC9137498

[20] Nielsen EI, Viberg A, Löwdin E, Cars O, Karlsson MO, Sandström M. 2007. Semimechanistic pharmacokinetic/pharmacodynamic model for assessment of activity of antibacterial agents from time-kill curve experiments. Antimicrob Agents Chemother 51:128–136. 10.1128/AAC.00604-06. 17060524 PMC1797646

[21] Greenwood D. 1975. The activity of polymyxins against dense populations of Escherichia coli. J Gen Microbiol 91:110–118. 10.1099/00221287-91-1-110. 172601

[22] Satola SW, Farley MM, Anderson KF, Patel JB. 2011. Comparison of detection methods for heteroresistant vancomycin-intermediate Staphylococcus aureus, with the population analysis profile method as the reference method. J Clin Microbiol 49:177–183. 10.1128/JCM.01128-10. 21048008 PMC3020420

[23] Sherman EX, Wozniak JE, Weiss DS. 2019. Methods to evaluate colistin heteroresistance in Acinetobacter baumannii. Methods Mol Biol 1946:39–50. 10.1007/978-1-4939-9118-1_4. 30798542 PMC6637766

[24] Liao W, Lin J, Jia H, Zhou C, Zhang Y, Lin Y, Ye J, Cao J, Zhou T. 2020. Resistance and heteroresistance to colistin in Escherichia coli isolates from Wenzhou, China. Infect Drug Resist 13:3551–3561. 10.2147/IDR.S273784. 33116674 PMC7553605

[25] Guyonnet J, Manco B, Baduel L, Kaltsatos V, Aliabadi MH, Lees P. 2010. Determination of a dosage regimen of colistin by pharmacokinetic/pharmacodynamic integration and modeling for treatment of G.I.T. disease in pigs. Res Vet Sci 88:307–314. 10.1016/j.rvsc.2009.09.001. 19945722

[26] Charretier Y, Diene SM, Baud D, Chatellier S, Santiago-Allexant E, van Belkum A, Guigon G, Schrenzel J. 2018. Colistin heteroresistance and involvement of the PmrAB regulatory system in Acinetobacter baumannii. Antimicrob Agents Chemother 62:e00788-18. 10.1128/AAC.00788-18. 29914966 PMC6125516

[27] Gefen O, Chekol B, Strahilevitz J, Balaban NQ. 2017. TDtest: easy detection of bacterial tolerance and persistence in clinical isolates by a modified disk-diffusion assay. Sci Rep 7:41284–41289. 10.1038/srep41284. 28145464 PMC5286521

[28] Nielsen EI, Khan DD, Cao S, Lustig U, Hughes D, Andersson DI, Friberg LE. 2017. Can a pharmacokinetic/pharmacodynamic (PKPD) model be predictive across bacterial densities and strains? External evaluation of a PKPD model describing longitudinal in vitro data. J Antimicrob Chemother 72:3108–3116. 10.1093/jac/dkx269. 28961946

[29] Bulitta JB, Yang JC, Yohonn L, Ly NS, Brown SV, D'Hondt RE, Jusko WJ, Forrest A, Tsuji BT. 2010. Attenuation of colistin bactericidal activity by high inoculum of Pseudomonas aeruginosa characterized by a new mechanism-based population pharmacodynamic model. Antimicrob Agents Chemother 54:2051–2062. 10.1128/AAC.00881-09. 20211900 PMC2863601

[30] Andersson DI, Hughes D. 2010. Antibiotic resistance and its cost: is it possible to reverse resistance? Nat Rev Microbiol 8:260–271. 10.1038/nrmicro2319. 20208551

[31] Vogwill T, MacLean RC. 2015. The genetic basis of the fitness costs of antimicrobial resistance: a meta-analysis approach. Evol Appl 8:284–295. 10.1111/eva.12202. 25861386 PMC4380922

[32] Ma K, Feng Y, Zong Z. 2018. Fitness cost of a mcr-1-carrying IncHI2 plasmid. PLoS One 13:e0209706. 10.1371/journal.pone.0209706. 30586457 PMC6306219

[33] Kluytmans J. 2017. Plasmid-encoded colistin resistance: mcr-one, two, three and counting. Eurosurveillance 22:30588. 10.2807/1560-7917.ES.2017.22.31.30588. 28797321 PMC5553061

[34] Tietgen M, Semmler T, Riedel-Christ S, Kempf VA, Molinaro A, Ewers C, Göttig S. 2018. Impact of the colistin resistance gene mcr-1 on bacterial fitness. Int J Antimicrob Agents 51:554–561. 10.1016/j.ijantimicag.2017.11.011. 29180279

[35] Yang QE, MacLean C, Papkou A, Pritchard M, Powell L, Thomas D, Andrey DO, Li M, Spiller B, Yang W, Walsh TR. 2020. Compensatory mutations modulate the competitiveness and dynamics of plasmid-mediated colistin resistance in Escherichia coli clones. ISME J 14:861–865. 10.1038/s41396-019-0578-6. 31896787 PMC7031280

[36] Mohamed AF, Cars O, Friberg LE. 2014. A pharmacokinetic/pharmacodynamic model developed for the effect of colistin on Pseudomonas aeruginosa in vitro with evaluation of population pharmacokinetic variability on simulated bacterial killing. J Antimicrob Chemother 69:1350–1361. 10.1093/jac/dkt520. 24474432

[37] Karvanen M, Malmberg C, Lagerbäck P, Friberg LE, Cars O. 2017. Colistin is extensively lost during standard in vitro experimental conditions. Antimicrob Agents Chemother 61:e00857-17. 10.1128/AAC.00857-17. 28893773 PMC5655071

[38] ISO (International Organization for Standardization). 2006. ISO 20776-1: 2006 Clinical laboratory testing and in vitro diagnostic test systems–Susceptibility testing of infectious agents and evaluation of performance of antimicrobial susceptibility test devices–Part 1: reference method for testing the in vitro activity of antimicrobial agents against rapidly growing aerobic bacteria involved in infectious diseases. International Organization for Standardization, 19.

[39] EUCAST. 2016. Recommendations for MIC determination of colistin (polymyxin E) as recommended by the joint CLSI-EUCAST Polymyxin Breakpoints Working Group. European Committee on Antimicrobial Susceptibility Testing: Växjö, Sweden. https://www.eucast.org/fileadmin/src/media/PDFs/EUCAST_files/General_documents/Recommendations_for_MIC_determination_of_colistin_March_2016.pdf.

[40] Mead A, Lees P, Mitchell J, Rycroft A, Standing JF, Toutain PL, Pelligand L. 2019. Differential susceptibility to tetracycline, oxytetracycline and doxycycline of the calf pathogens Mannheimia haemolytica and Pasteurella multocida in three growth media. J Vet Pharmacol Therap 42:52–59. 10.1111/jvp.12719. 30267412

[41] Sidhu P, Landoni M, Aliabadi M, Toutain P-L, Lees P. 2011. Pharmacokinetic and pharmacodynamic modelling of marbofloxacin administered alone and in combination with tolfenamic acid in calves. J Vet Pharmacol Ther 34:376–387. 10.1111/j.1365-2885.2010.01247.x. 21091727

[42] Dorey L, Hobson S, Lees P. 2016. Activity of florfenicol for Actinobacillus pleuropneumoniae and Pasteurella multocida using standardised versus non-standardised methodology. Vet J 218:65–70. 10.1016/j.tvjl.2016.11.004. 27938711

[43] Chauzy A, Ih H, Jacobs M, Marchand S, Grégoire N, Couet W, Buyck J. 2020. Sequential time-kill, a simple experimental trick to discriminate between pharmacokinetics/pharmacodynamics models with distinct heterogeneous subpopulations versus homogenous population with adaptive resistance. Antimicrob Agents Chemother 64:e00788-20. 10.1128/AAC.00788-20. 32513802 PMC7526804

[44] Beal SL. 2001. Ways to fit a PK model with some data below the quantification limit. J Pharmacokinet Pharmacodyn 28:481–504. 10.1023/A:1012299115260. 11768292

[45] Pelligand L, Lees P, Sidhu PK, Toutain P-L. 2019. Semi-mechanistic modeling of florfenicol time-kill curves and in silico dose fractionation for calf respiratory pathogens. Front Microbiol 10:1237. 10.3389/fmicb.2019.01237. 31244793 PMC6579883

[46] Mouton JW, Dudley MN, Cars O, Derendorf H, Drusano GL. 2005. Standardization of pharmacokinetic/pharmacodynamic (PK/PD) terminology for anti-infective drugs: an update. J Antimicrob Chemother 55:601–607. 10.1093/jac/dki079. 15772142

